# A novel tamponade agent for management of post partum hemorrhage: adaptation of the Xstat mini-sponge applicator for obstetric use

**DOI:** 10.1186/s12884-017-1373-x

**Published:** 2017-06-13

**Authors:** Maria I. Rodriguez, Jeffrey T. Jensen, Kenton Gregory, Mary Bullard, Paul Longo, Jerry Heidel, Alison Edelman

**Affiliations:** 1 Section of Family Planning, Department of Obstetrics and Gynecology, Oregon Health & Science University, 3181 SW Sam Jackson Park Rd, Portland, OR 97239 USA; 2 Center for Regenerative Medicine, Oregon Health & Science University RevMedx Inc, Wilsonville, USA; 3 Oregon State University, Corvallis, USA

**Keywords:** Postpartum hemorrhage, Low resource setting, Uterine tamponade

## Abstract

**Background:**

Although uterine tamponade is an effective treatment for postpartum hemorrhage (PPH), current methods have key limitations in their use, particularly in low resource settings. The XStat™ Mini Sponge Dressing (MSD) is approved for the management of non-compressible wounds in the battlefield/trauma setting. The MSD applies highly compressed medical sponges capable of stopping high-flow arterial bleeding within seconds. The objective of our study was to adopt the MSD for use in managing PPH.

**Methods:**

We performed desktop testing using a uterine model with pressure sensors to compare key design elements of the obstetrical prototype MSD (fundal pressure achieved, reduction in fluid loss, time to deploy, and time to remove) with alternativetechniques (uterine packing, balloon tamponade). To evaluate safety, we delivered the fetus of pregnant ewes by cesarean section and used the prototype to deliver the MSD into one uterine horn, and closed the hysterotomy. We followed the clinical recovery of animals (*n* = 3) over 24 h, and then removed the reproductive tract for histologic evaluation. To evaluate late effects, we surgically removed the MSDs after 24 h, and followed the clinical recovery of animals (*n* = 6) for an additional seven days before tissue removal.

**Results:**

The obstetrical prototype has a long tapered delivery system designed to be deployed during vaginal examination, and administers three times the volume of the approved MSD trauma bandage. The MSD are deployed within a mesh bag to facilitate removal by vaginaltraction. On desktop testing, the MSD resulted in the highest average fundal pressure (113 mmHg), followed by the MSD bag device (85.8 mmHg), gauze packing (15.5 mmHg), and the uterine balloon (8.2 mmHg). The MSD bag test group achieved the largest fluid flow reduction of −74%, followed by gauze packing (−55%), MSD (−35%), and uterine balloon (−19%). Animal testing demonstrated good uterine fill with no evidence of adverse clinical recovery, uterine trauma or infection at 24 h, or up to 7 days following device removal.

**Conclusion:**

We adapted a highly effective trauma dressing and applicator for use in the treatment of severe PPH. Preliminary desktop and animal testing provide a basis for initial clinical trials in women.

**Electronic supplementary material:**

The online version of this article (doi:10.1186/s12884-017-1373-x) contains supplementary material, which is available to authorized users.

## Background

Postpartum hemorrhage (PPH) is the leading cause of maternal mortality in low-income countries [1, 2]. The majority of these deaths occur outside the health care system, thus an intervention that can be used in any setting with minimal training could save lives. Primary PPH is typically defined as a blood loss of 500 ml or more within 24 h of a birth [1]. Uterine atony, or the ineffective contraction of the uterus, is responsible for the bulk of PPH cases. The majority of all deaths related to PPH will occur within 24 h following delivery in settings, where operative measures are not readily available. Nearly all of these deaths could be prevented by swift intervention with a series of non-operative and operative measures [3].

Recently, much effort has been devoted to increasing the access to and training in low-cost highly effective uterotonics such as misoprostol [1, 4]. However, this treatment is not always effective, and women with hemorrhage that do not respond to uterine massage or uterotonics must be transported to the hospital for further care. Hospitals are often remote, poorly equipped, and transport is not easily accomplished. For women in these settings experiencing severe hemorrhage, temporizing measures are needed.

Uterine tamponade is both an effective stabilizing treatment and an intermediate measure that can be implemented for reducing blood loss while preparing for definitive surgery [5–9]. Strategies for implementing uterine tamponade include uterine packing with sterile gauze, inflated foley catheters, condom catheters, and silicone obstetrical balloons [3, 10, 11]. Although case series data demonstrate the efficacy of these methods, none is ideal for use in the low resource setting. Effective packing with adequate amounts of sterile gauze is not readily accomplished even by a highly skilled provider in a patient without anesthesia. Deployment of a balloon catheter, while more effective, is a higher-level procedure that requires having several components immediately available (e.g balloon or condom, sterile water, syringe, ultrasound). Use of some of these devices in low resource settings is also limited by their cost.

**Fig. 1:**
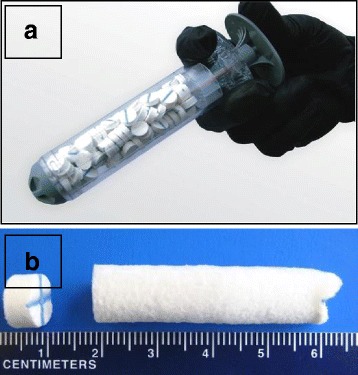
The MSD trauma applicator. **a**. MSD mini-sponge dressing with applicator. **b**. Compressed and expanded mini-sponge

An innovative bandage, the XStat™ mini sponge dressing (MSD; RevMedx, Inc. 25,999 SW Canyon Creek Rd., Suite C, Wilsonville, OR 97070), has proven successful in the acute cessation of traumatic non-compressible bleeding analogous to PPH [12]. The MSD is capable of stopping high-flow arterial bleeding (i.e., >1.5 L/min) within seconds, without intrinsic or external compression [12]. This device utilizes pre-packaged, environmentally stable, highly compressed medical sponges administered by a light-weight applicator similar to a large syringe (Fig. 1). In a bleeding wound, the mini-sponges rapidly absorb blood and expand filling the wound cavity and providing a nearly immediate hemostatic effect. In a study comparing the MSD to a leading hemostatic gauze in a lethal porcine subclavian hemorrhage model, the MSD provided dramatic improvements in survival and hemostasis 60 min after injury, with a large reduction in blood loss and treatment time [12]. The MSD has been approved by the United States Food and Drug Administration for use on the battlefield and in civilian trauma settings [13].

The objective of our study was to adopt the MSD for use in managing PPH. We had two specific aims. The first was to modify the trauma applicator to allow for uterine deployment and removal vaginally. The second was to conduct desktop and animal testing to demonstrate both proof of concept and safety.

## Methods

The first phase of our research involved developing a prototype of an obstetrical applicator and conducting in vitro testing (desktop testing) for preliminary feedback. The objective of this phase was to modify the trauma applicator to be suitable for obstetric use, and to develop a method to remove the mini-sponges without surgery. The second phase of our research involved testing the obstetrical applicator in an animal model. We sought to demonstrate that the mini-sponges would fill a uterus without any evidence of damage to the uterus (trauma or infection). The research was conducted following approval of the institutional animal care committee at Oregon Health & Science University and Oregon State University. Animals were obtained from a commercial source.

### Development of an obstetrical applicator: clinical design review

To develop the obstetrical prototype, our team had several group design reviews to determine what device modifications would be needed to successfully adapt the trauma applicator. Our team included engineers with product development expertise, and obstetricians. We discussed normal human anatomy including determining average distances from the vaginal introitus to the cervix, and cervix to uterine fundus, as well as standard uterine volumes and cervical dilation immediately postpartum. We discussed what anatomical changes would be expected in the first 72 h postpartum in terms of uterine and cervical size. This was important to consider as we debated how to adapt the device so that the mini-sponges could be removed by vaginal traction, rather than a more invasive procedure (removal of the mini-sponges with the trauma applicator is by exploratory surgery). We agreed to remove the sponges through a simulated cervix, a non-distensible two centimeter opening to ensure that the sponges could readily be removed in a clinical setting. We considered options such as deploying the sponges in a bag, or linking them on a string to meet this goal.

### Development of an obstetrical applicator: desktop testing

**Fig. 2:**
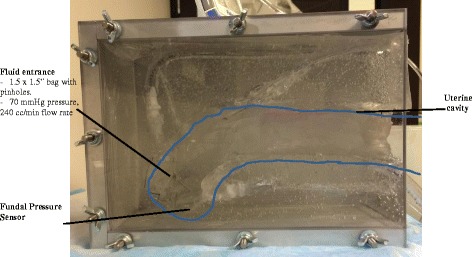
PPH Uterine model for Desktop testing

To augment the clinical review feedback, information on the mechanical properties of the device in a uterine model was obtained, to finalize input to the prototype design. This included collecting information on aspects such as mini-sponge flow and pressures achieved. We wanted to compare some of the basic properties of our device to what is currently used in uterine tamponade (uterine balloon, and packing with gauze) the MSD in different formats (the standard loose pellets as used in the trauma applicator and MSD pellets in a fine mesh bag designed to allow full sponge expansion but to contain the sponges for easy removal by a vaginal exam). To achieve this goal, we built a desktop model of a postpartum uterus, which contained sensors to record pressure (Fig. 2).

**Fig. 3:**
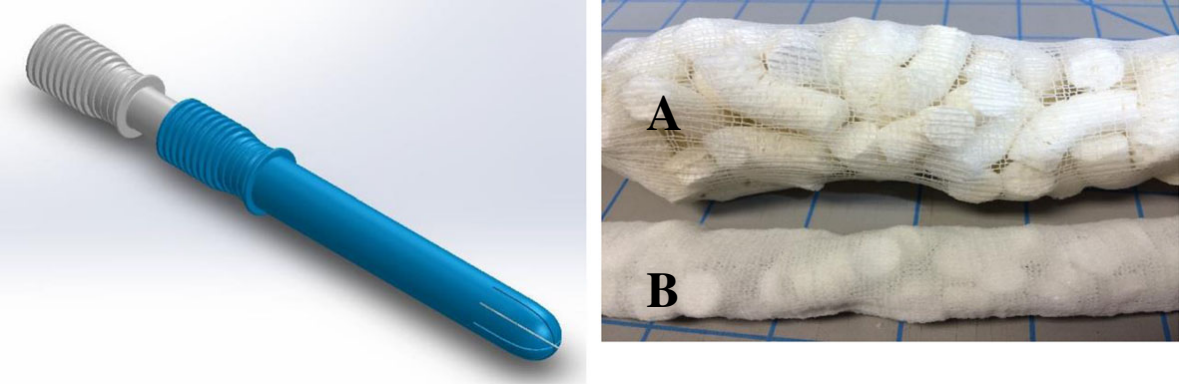
Revised design of obstetrical applicator and sponge removal system. “Sponges in a bag” system for ease of vaginal removal. **a** Expanded sponges following removal. **b** Dry sponges pre-deployment

A 350cm^3^ post-partum uterine cavity with a 4 cm diameter opening was molded out of gelatin (Fig. 3). Two, 1 × 1 in. pressure sensors were located in the uterus, and connected to a National Institutes of Health data acquisition system for continuous pressure data collection. 45% glycerol was plumbed into the uterus with a hydrostatic pressure head equivalent to 73 mmHg (39 in. high). Fluid entered the model uterus through a 1.5 × 1.5 in. bag with pinholes. The unimpeded fluid flow rate was 240 cm^3^/min. The uterus was filled with 100 cm^3^ of 45% glycerol solution, and then the test material was inserted. The pressure measurements were continuously recorded via Labview software, and the fluid flow rate was measured gravimetrically.

We compared the following metrics: application time (seconds); reduction in fluid flow rate (%); fundal pressure achieved (mmHg); and removal time (seconds). We analyzed differences in these outcomes among two different formulations of the MSD (the sponges as delivered in the trauma applicator, and the mini-sponges in a medical grade bag) with two materials commonly used for uterine tamponade. These included gauze and a uterine balloon, assembled with a Foley catheter and condom.

### Animal testing

The objective of animal testing was to assess whether the prototype obstetric MSD could fill a postpartum uterine cavity with minisponges and maintain contact pressure over 24 h without causing adverse systemic or local tissue effects. Two phases of animal testing occurred, with the first three animals followed for three days, and an additional six animals followed for seven days.

We conducted a literature search to determine the best animal model for PPH. Currently there is no true postpartum animal model to study PPH. However, pregnant ewes have a similarly distensible uterus with a very vascular placenta similar to humans [14].

Ewes were obtained from a commercial source. We selected ewes between day 59–150 of gestation because the size of the uterine horn containing the fetus is comparable in size (volume) as that of a apostpartum human uterus [15]. The sheep uterus also contains multiple horns, or different sections. This allowed us to treat one horn with the MSD and to use another horn as a control.

We designed our animal experiments to mimic the expected clinical scenario for use of this applicator intrauterine placement for up to 24 h for stabilization and potential transfer of care, followed by removal. To evaluate the short term endpoint of clinical tolerance and histologic effects of sponge placement, animals were sacrificed at 24 h and the uterus removed with the MSD in situ. To evaluate longer term effects of treatment, we removed sponges by hysterotomy after 24 h, and then observed the animals for any untoward effects (e.g. bleeding, pain, infection) post-removal for 7 days prior to necropsy. Animals were humanely euthanized with an overdose of a pentobarbital euthanasia solution. After gross findings were recorded, full thickness representative samples of the treated and untreated uterine horns were collected for histologic evaluation. These samples included glandular endometrium as well as the non-glandular “caruncles,” the latter being the sites of placental attachment in the ovine uterus. In brief, the specimens were fixed in 10% neutral buffered formalin and processed using routine methods. Four micron sections, stained with hematoxylin and eosin, were examined by veterinary pathologists at the Oregon Veterinary Diagnostic Laboratory, Oregon State University in a blinded fashion. The protocol for animal testing is summarized in Table 1.

**Table 1:** Protocol for testing the Mini Sponge Dressing in a ewe model

Step 1	Complete blood count (CBC) obtained prior to abdominal incision.
Step 2	General anesthesia was induced and a laparotomy incision was made.
Step 3	Uterus evacuated by hysterotomy.
Step 4	Following evacuation of fetus, the umbilical cord was unclamped and the placentomes (sections of the sheep placenta) cut at the base and curetted to cause bleeding for approximately 60 s. Placentomes are composed of the maternal caruncle and fetal cotyledon. Several entire placentomes were cut off at their bases to promote hemorrhage. The endometrium/myometrium was gently curetted at each extirpated placentome site to increase hemorrhage.
Step 5	The hysterotomy incision was closed in the usual fashion until only a 1–2 cm opening remained.
Step 6	The MSD obstetrical applicator was then placed through the partially closed hysterotomy (to mimic the cervix) and the sponges deployed to fill the uterus.
Step 7	The remaining aspect of the hysterotomy incision was then closed, followed by the abdominal skin incisions using standard technique.
Step 8	Anesthesia was then reversed.
Step 9	All experimental ewes were treated with non steroidal anti-inflammatory drugs for pain (flunixin meglumine (Banamine)) at the conclusion of surgery and 24 h later. Animals were carefully monitored throughout this period for signs or symptoms of pain, fever and bleeding. If signs of discomfort (reluctance to rise, grinding of teeth, head pressing, loss of appetite, fever) were still evident the day after surgery, additional analgesia (meloxicam, 1 mg/kg q24 h po) was provided.
Step 10	At 24 h post-operative, a second CBC was obtained.
Step 11	For the first three animals, 24 h after the initial surgery, the animal was euthanized, and the entire uterus removed for tissue analysis, with the sponges left in place. For the remaining six animals, 24 h after the initial surgery, the sponges were removed by laparotomy. The animal was then observed for an additional six days. At one week following initial deployment, the animal was euthanized, and the entire uterus removed for tissue analysis, with the sponges left in place.
Step 12	Histologic analysis of the uterus was then performed to evaluate for signs of infection or trauma from the sponges.

## Results

### Clinical design

We developed an obstetrical prototype of this device suitable for use in the management of PPH in low-resource settings. The clinical design review sessions produced the following recommended device modifications:

One applicator should ideally contain enough pellets to fill a volume of 350–500 cm^3^.

The applicator should be at least 15 cm in length, to allow adequate distance to reach from the vaginal opening into the lower uterine segment.

The device should be able to be placed without direct visualization of the cervix, by threading the applicator over a vaginal hand.

The diameter of the applicator should be less than 3 cm to allow for comfortable vaginal placement and navigating the cervix.

A soft rounded tip on the applicator is needed to minimize risk of vaginal or uterine trauma.

Ideally the applicator should be able to be deployed using one hand.

Removal of the mini-sponges must be by a vaginal exam, through a two centimeter cervix, without need for a more invasive procedure.

**Fig. 4:**
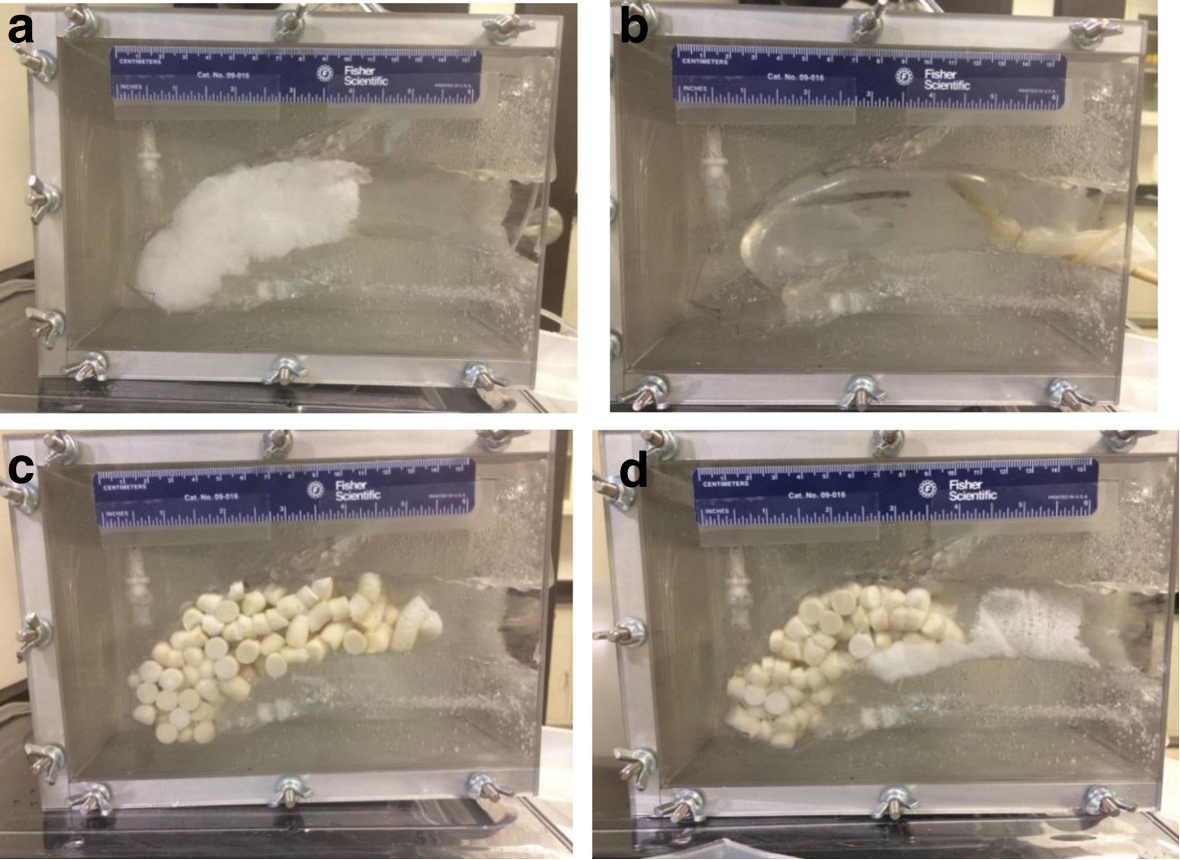
Representative pictures of test materials inserted into PPH uterine model. Pictures **a**, **b**, **c**, and **d** are Gauze, UBT, MSD, and MSD bag, respectively

The obstetrical applicator delivers three times the volume of MSD as the trauma applicator, and is considerably longer (Fig. 4). It is designed to be deployed vaginally, with the tip placed transcervically in the lower uterine segment. The MSD are released in mesh bags with long transcervical strings to facilitate subsequent removal by a vaginal exam.

### Desktop testing

Representative pictures of the uterine cavity illustrate how well the test material conformed to theuterine cavity (Fig. 4). Both formulations of the MSD mini-sponges (loose or in a bag) were uniformly distributed throughout the uterine model. The test results, including fundus pressure, flow reduction, application time, and removal time, are summarized for each group in Table 2. The MSD material resulted in the highest average fundal pressure of 113 mmHg, followed by the MSD bag (85.8 mmHg), gauze (15.5 mmHg), and the uterine condom balloon (8.2 mmHg). All groups were found to be significantly different from each other, with the exception of gauze and the uterine condom balloon.

**Table 2:** Pressure and flow reduction of wound dressings in postpartum hemorrhage uterine model

Group	n	Fundal Pressure (mmHg)	Flow reduction (%)	Application Time (sec)	Removal Time (sec)
Gauze	8	15.5 (8.0)	−55 (10)	59 (10)	9 (2)
Uterine Balloon	8	8.2 (10.4)	−19 (17)	194 (73)	18 (8)
MSD	8	113.0 (28.6)	−35 (9)	11 (2)	266 (85)
MSD bag	8	85.8 (29.0)	−74 (18)	12 (3)	10 (2)

The MSD bag test group achieved the largest fluid flow reduction of −74%, followed by gauze (−55%), MSD (−35%), and uterine balloon (−19%). The application time of the test materials ranged from seconds (11 and 12 s for MSD and MSD bag) to several minutes (~3 min for the uterine balloon). The removal time varied similarly from ~10s for both gauze and the MSD bag to ~4 min for MSD (Table 2).

As seen in the representative pictures of each test group in Fig. 4, the uterine balloon could not reach the fundus, stopping typically about a 1 cm away from the fundus. This resulted in the test group achieving a lower pressure and fluid flow reduction compared to the other control group, gauze. When the uterine balloon did reach the fundus, it tended to move after being placed as a result from the fluid flow pushing out the balloon. The MSD bag and the MSD test groups had similar pressure vs time curves in which the pressure slowly increased within the first minute after placement (due to continued activation of the compressed sponges) and then maintained that pressure until the sponges were removed from the uterus.

### Animal testing

A total of nine ewes, with gestations ranging from 127 to 159 days were enrolled. Mean uterine dimensions following evacuation of the fetus was 23.8 cm long and 12 cm wide. An average of two obstetrical applicators was needed to fill the uterus. In Phase 1, surgery was completed on three animals. Good uterine fill was noted on deployment, and on re-inspection 24 h postpartum. In fact, considerable expansion of the sponges occurred after the specimen was opened, demonstrating a residual capacity to expand in a clinical situation where uterine tone might vary. Moreover, while the sponges demonstrated filling and a capacity for additional expansion, the pressure was not excessive; although the hysterotomy incision was closed under tension, the sponges did not compromise the closure. On gross inspection, the control horn (untreated) visually had more blood clots present than the treatment horn. In Phase 2, six pregnant ewes with gestations ranging from 127 to 159 days were enrolled. Preoperative hematocrits averaged 38% (range 35.9–44.6%). In all subjects, the sponges were easily deployed into the uterus with good uterine fill. At reoperation 24 h later, all six females were found to have the treated uterine horn firmly distended with an intact hysterotomy. To simulate transcervical removal, we inserted a non-distensible two centimeter ring through the prior hysterotomy, and delivered the MSD bags through this.

Post-operatively, the females recovered without complication until scheduled necropsy at 24 h (Phase 1) or 7 days following MSD removal (Phase 2). One sheep had a poor appetite, and was given a second dose of anti-inflammatory medication on post-operative day 2. At the time of hysterectomy, no acute intra-abdominal findings suggesting infection or trauma were noted. Gross inspection of the excised reproductive structures revealed them to be without abnormality.

**Fig. 5:**
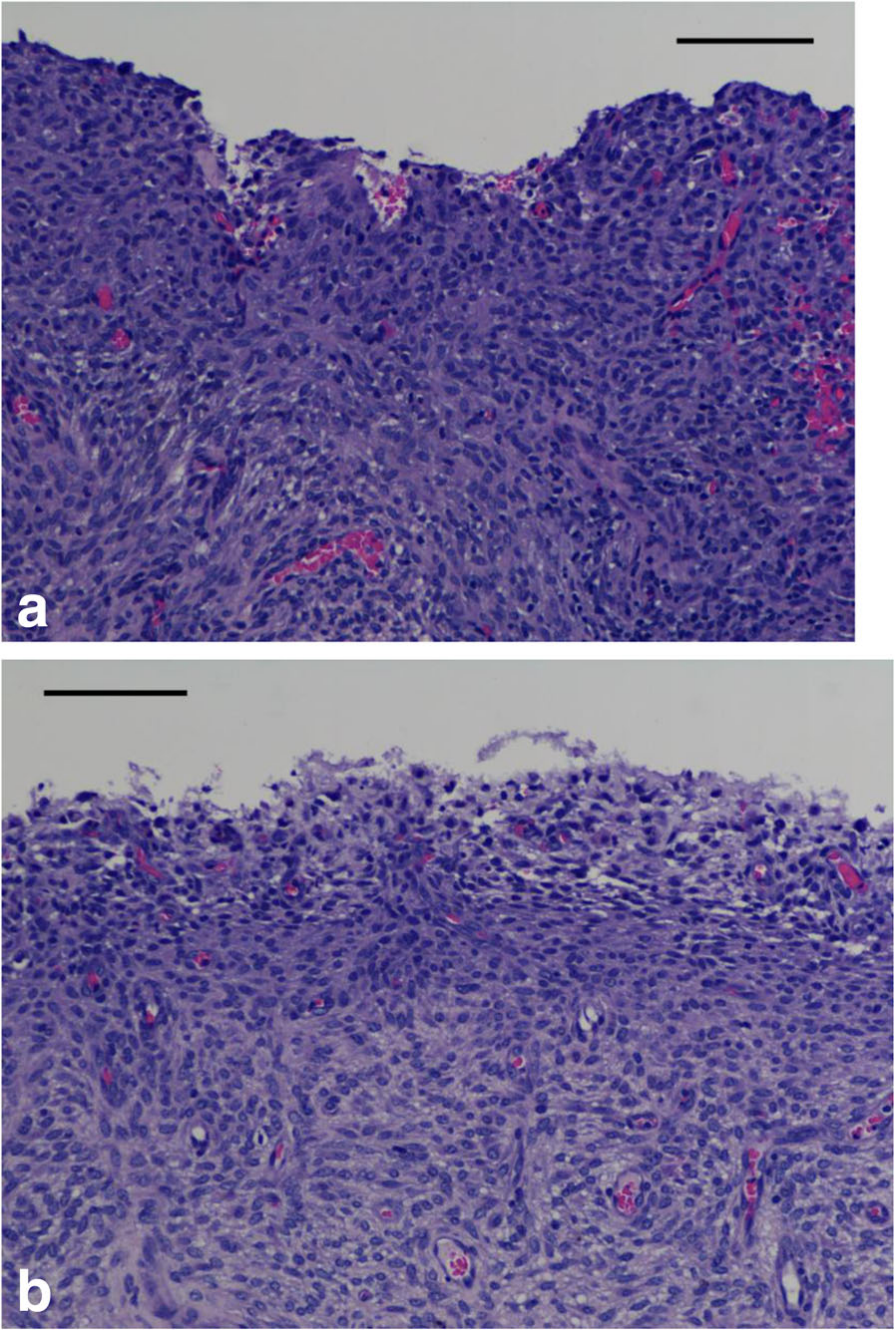
Histological comparison of control and treated uteri 7 days following application. **a** Section of uterus treated with MSD. The non-glandular endometrial caruncle, site of placental attachment, harbors rare lymphocytes and neutrophils within the submucosal layer, a reaction to the experimental manipulation. The mucosal epithelium is lost due to experimental manipulation. No evidence of infection or fibrosis is present. Bar = 100 μm. **b** Control uterus shows no histological difference from treated tissue. Bar = 100 μm

Inspection of serial sections of the control and treated uterine horns supported the visual review. Sections from the treated horn were similar to control: no microscopic findings suggestive of infection or trauma were noted (Fig. 5).

## Discussion

We have developed an obstetrical prototype and preliminary desktop and animal testing yield promising results. The MSD appears to be a safe and effective option for treating PPH in an animal model. It may have the potential to be a useful agent for treating severe PPH in a low resource setting.

What we learned from the desktop testing was that the MSD mini-sponges, both loose and in a bag, conformed well to the uterine curvature when compared with other therapies used for PPH. This suggests that the sponges flow well to fill the uterine cavity, and that they would be well positioned to exert pressure on the uterine wall and stop hemorrhage, an essential first step in managing bleeding. We then looked at how well the different treatments worked in delivering pressure, the means by which bleeding is stopped. When we examined measures of fundal pressure, we learned that the MSD in both formulations delivered the highest level of pressure, well above what is seen with comparative treatments such as packing with gauze or the uterine balloon kit.

The desktop testing indicates that both delivery forms of the MSD are not only able to reach the uterine fundus, but also to maintain higher levels of pressure than materials that are currently the standard of care for controlling PPH. Holding pressure, especially at the uterine fundus, is essential to stopping the most common cause of severe PPH. This is a promising finding—suggesting that the MSD may offer an advantage over existing materials in the treatment of PPH.

We tested the device in an ovine model to see if the MSD would fill the uterine cavity and maintain tamponade over a 24 h period. The animal studies were also used to evaluate whether the sponges in a bag could be removed through a simulated two centimeter cervix. Our findings suggest that the MSD in a bag would allow for removal through a contracted cervix postpartum in a human model, an important adaptation for obstetric use. We also evaluated for measures of safety, such as histologic evidence of uterine trauma or infection. All sheep did well without signs of infection. The reproductive tracts of all six females were removed at necropsy, and there was no gross or histologic evidence of endometritis.

A key limitation of our research has been the absence of a validated animal model for studying postpartum hemorrhage in humans. After review of existing literature, the sheep model was identified as the best model for studying uterine filling and tissue effects. However, in our risk management plan, we had identified the risk that the sheep uterus model may have a much lower volume of bleeding than seen in humans. This is in fact, what was observed during the first three animal surgeries: the sheep uterus, due to different tissue and vascular properties had minimal blood loss. This limitation affects all products being developed to treat PPH. We are thus unable to assess for effectiveness of treating PPH in an animal model. We do however, have compelling data that the MSD effectively stops arterial hemorrhage (non-uterine) in a porcine model [16]. With the existing animal data from both models on safety, feasibility, and effectiveness, the next step would be a small study in postpartum women.

We also consider as a limitation the lack of end user feedback (beyond our immediate study team) on ease of use of the MSD obstetrical applicator. Obtaining and including input on the design from midlevel providers, and organizations that we would want to work with to disseminate the device during scale up was identified as a priority for the next phase of work.

## Conclusion

PPH remains a common cause of maternal morbidity and mortality, particularly in low resource settings. There is an urgent need to develop innovative strategies that can be used by care providers with minimal training and equipment. The MSD has impressive efficacy in stopping non compressible arterial hemorrhage from battlefield injuries, a setting that is clinically analogous to women experiencing PPH [12].

Animal testing demonstrates that the MSD can be deployed postpartum, safely remain in place for 24 h, and then be removed without causing uterine damage. This suggests the device would be safe and effective for treating PPH in women. Next steps for this work include obtaining regulatory approval for use in postpartum women, and a Phase 1 clinical trial to demonstrate feasibility, safety, and acceptability. We will first assess the ease of placement and removal in a small cohort of women undergoing an uncomplicated birth or second trimester abortion. We then plan to study the device used in the emergent settings for women refractory to medical therapies where surgery is being considered.

## Additional file

Additional file 1: Desktop testing of postpartum hemorrhage models. Raw data from desktop testing of postpartum hemorrhage models. (XLSX 14 kb)

## Abbreviations

FDA: Food and Drug Administration
MSD: Mini-sponge dressing
PPH: Postpartum Hemorrhage
UBT: Uterine Balloon Tamponade
